# Prenatal ultrasound diagnosis and prognosis of fetus with isolated filar cyst: a retrospective analysis

**DOI:** 10.3389/fmed.2024.1304803

**Published:** 2024-01-22

**Authors:** Song Chen, Peng Tu, Lan Mu

**Affiliations:** Department of Ultrasound, Chongqing Health Center for Women and Children, Women and Children’s Hospital of Chongqing Medical University, Chongqing, China

**Keywords:** filar cysts, ultrasound, prenatal diagnosis, prognosis, fetus

## Abstract

**Objective:**

This study aimed to investigate the prenatal ultrasonographic diagnosis and prognosis of fetuses with isolated filar cysts (FCs).

**Methods:**

The ultrasonographic features, reasons for missed diagnosis, and prognosis of eight isolated FCs diagnosed using ultrasound were analyzed retrospectively through follow-up.

**Results:**

Eight isolated FCs showed round or fusiform cystic anechoic areas at the end of the conus medullaris. Among them, six cases were prenatally diagnosed and the other two cases were diagnosed after birth. Of the six cases diagnosed prenatally, four (66.7%) disappeared during pregnancy, and the shortest time to disappearance was 1 month after the first diagnosis. All patients were followed up without any clinical symptoms or functional abnormalities.

**Conclusion:**

Isolated FCs may exhibit physiological variations that disappear spontaneously during pregnancy and usually have no clinical symptoms. They are usually benign and have a good prognosis. Ultrasonography is helpful for the diagnosis and follow-up of FCs.

## 1 Introduction

Human spinal cord development goes through three main stages: (1) neurulation, (2) tube formation, and (3) degenerative differentiation. Both the conus medullaris and terminal filaments are formed in the last stage. With rapid growth of the spine, the terminal filament at the end of the spinal cord is elongated. The pathogenesis of filar cysts (FCs) remains unclear and may be related to excessive pipeline or incomplete degenerative differentiation during spinal cord development (1, 2).

Filar cysts (FCs) rarely occur in the spinal canal. They typically present as anechoic structures at the ends of the conus medullaris. They are usually found by ultrasound or MRI in young infants. However, FCs are hidden during the fetal period, and lesions are small and easy to miss during diagnosis. Reports on the prenatal ultrasound diagnosis of this disease are rare (3–5). This study retrospectively analyzed eight cases of isolated FCs diagnosed by ultrasound, focusing on the characteristics of ultrasound images, reasons for missed diagnosis, and prognosis.

## 2 Materials and methods

### 2.1 Materials

We retrospectively analyzed eight cases of isolated FCs diagnosed by ultrasound at our hospital from January 2020 to October 2022. These included six cases of prenatal diagnosis, pregnant women with gestational weeks of 22–37 weeks, aged 25–33 years, and no history of adverse pregnancy. Two cases were diagnosed after birth and missed prenatal ultrasound. This study was approved by the ethics committee of Chongqing Health Center for Women and Children (No. 2023069).

### 2.2 Methods

The prenatal ultrasonic diagnostic instruments used were GE Voluson E8 and E10, and the frequency of the two-dimensional abdominal convex array probe was 2.0–8.0 MHz. A Philips Elite neonatal ultrasonic examination instrument was used and the frequency of the high-frequency probe was 2.0–8.0 MHz. The “Guidelines for Prenatal Ultrasound Screening” and “Guidelines for Chinese Pediatric Ultrasound Examination” were followed to scan the spine with a longitudinal section and transverse section. In the suspected cases, the echogenic structure and position relative to the conus medullaris were further examined. After the first prenatal ultrasound discovery, three cases underwent MRI re-examination, and all prenatal cases underwent ultrasound re-examination every 2–4 weeks during pregnancy. All patients were followed up for at least 8 months after birth, and the longest follow-up duration was up to 2 years of age.

## 3 Results

### 3.1 Isolated diagnosed FCs

There were eight cases in this group: six cases were prenatally diagnosed, and two cases were missed prenatally. No abnormalities were detected by ultrasound; four patients underwent non-invasive DNA examination without abnormalities, and one patient underwent amniocentesis without abnormalities. The risk for Down syndrome in the remaining patients was low. Pregnant women in this group had no history of adverse pregnancies or childbirth and no pregnancy complications.

### 3.2 Ultrasound images and MRI manifestations of FCs

**FIGURE 1:**
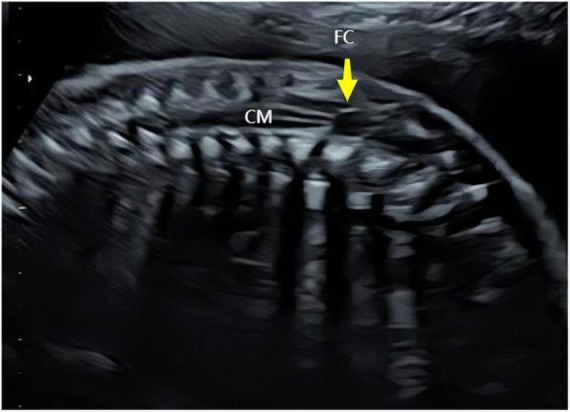
Longitudinal sections of the prenatal ultrasound of the spine. Yellow arrows indicate cysts of the terminal filament (FC) and conus medullaris (CM).

**FIGURE 2:**
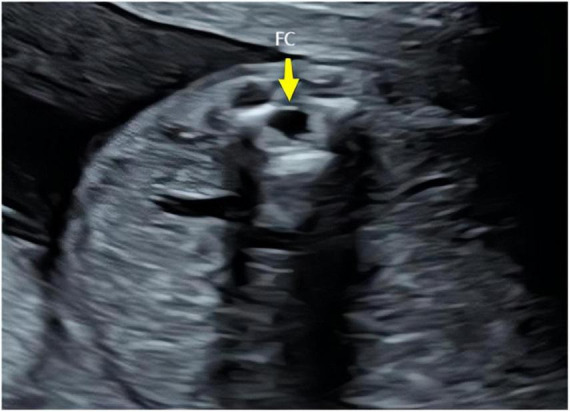
Transverse sections of the prenatal ultrasound of the spine. FC, terminal filament.

**FIGURE 3:**
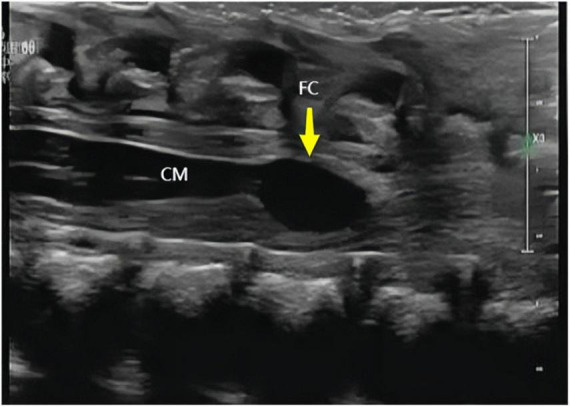
Longitudinal section of the spine obtained using neonatal high-frequency ultrasound. Yellow arrows indicate cysts of the terminal filament (FC) and conus medullaris (CM).

The ultrasound findings of the eight cases of isolated FCs were as follows: (1) a cystic anechoic area at the end of the conus medullaris, mostly located at the L2–L4 level in the lumbosacral spinal canal; (2) the cystic anechoic area had clear boundaries and a regular shape, and the longitudinal section showed an oval or fusiform, round cross-section, with no peripheral and internal blood flow signals; and (3) the position of the conus medullaris was normal (Figures 1–3). In this group of cases, the smallest cyst was 0.44 cm × 0.32 cm, the largest was 1.08 cm × 0.72 cm, and the average size was 0.69 cm × 0.40 cm.

Three patients with FCs underwent MRI examinations, and imaging showed localized T2 signals in the spinal canal at the end of the conus medullaris, which was slightly higher than that in the surrounding cerebrospinal fluid (Supplementary Figure 1).

### 3.3 Follow-up results of FCs

Ultrasound reexamination was performed every 2–4 weeks for six cases of prenatal diagnosis in this group. Among these, the FCs disappeared during pregnancy in four cases. The shortest disappearance time was at 28 weeks of gestation, 1 month after the first diagnosis. The FCs did not disappear during pregnancy in the other two cases. Follow-up ultrasonographic examinations were performed on all neonates on the first postpartum day. These findings were similar to those observed before delivery. The cysts were re-examined 1 month later, and a reduced cyst size was observed. Prenatally and postnatally diagnosed cases were followed up. None of the patients had clinical symptoms after birth, and there were no abnormalities in movement or defecation, as shown in Table 1.

**TABLE 1:** Ultrasound examination and follow-up results of FCs.

Case	Age	Gestational weeks	FCs size (cm)	FCs location	Chromosome examination	Follow-up results
1	31	23	0.60 × 0.51	L3	Amniocentesis test normal	Disappearance during pregnancy, 30 days after the first diagnosis
2	26	27	0.44 × 0.32	L3	Down’s screening low risk	Disappearance during pregnancy, 36 days after the first diagnosis
3	28	23	0.60 × 0.36	L2-3	Down’s screening low risk	Disappearance during pregnancy, 42 days after the first diagnosis
4	25	23	0.66 × 0.19	L3-4	Down’s screening low risk	Disappearance during pregnancy, a 52 days after the first diagnosis
5	33	35	0.81 × 0.53	L1-2	NITP low risk	Cyst size reduced after birth, no clinical symptoms during the follow-up period
6	25	37	1.08 × 0.72	L1-3	NITP low risk	Cyst size reduced after birth, no clinical symptoms during the follow-up period
7	30	1 day after birth	0.66 × 0.32	L3	Down’s screening low risk	Missed prenatally, no clinical symptoms during the follow-up period
8	31	2 days after birth	0.63 × 0.31	L3	NITP low risk	Missed prenatally, no clinical symptoms during the follow-up period

FCs, filar cysts; NITP, non-invasive prenatal testing.

## 4 Discussion

### 4.1 Prognosis and missed diagnosis analysis of solitary filum terminale cyst

All children had a good prognosis, and these results were consistent with those reported in two studies on the prenatal diagnosis of FCs (3, 4). Specifically, Wu et al. reported three cases of FCs diagnosed using prenatal abdominal ultrasonography. They found that the FCs spontaneously disappeared from the uterus. The shortest disappearance time was 4 weeks. Youssef et al reported two cases of FCs. One case was discovered using transvaginal ultrasound at 20 weeks of gestation, and there were no successful ultrasound follow-up records owing to fetal position factors. Another case was found using transabdominal ultrasound at 40 weeks of gestation, but there was a lack of postnatal ultrasound follow-up results. However, both patients remained healthy during the 6-month follow-up survey without clinical symptoms.

In this group of cases, the FCs did not disappear during pregnancy in two cases, the FCs were found after birth in two cases, and the subsequent follow-up decreased, which is consistent with several reports on postnatal FCs in infancy (5–7). Seo et al. retrospectively analyzed 396 infants with lumbosacral skin defects. They found that 56 (14.1%) patients had FCs, eight of which were combined with lipoma terminale, and six patients were withdrawn from the study. After 3–4 years, the FCs decreased in size or disappeared in 28 cases, they were reduced by non-surgical treatment in 3 cases, they did not change in size but the patient had no clinical symptoms in 17 cases, and they increased in size and underwent surgical treatment in 2 cases, both of which involved FCs combined with lipomas. No tumor-like tissue was observed in the resected cysts. Irani et al. performed a lumbar spine ultrasound in a case-control study of 644 infants less than 8 months of age and found that FCs occurred in 78 of the 644 infants (11.8%) and were negatively correlated with age. In the follow-up study, the children were able to turn over, crawl, and walk, with no statistically significant differences between the case and control groups. Choi’s study included 230 infants under 6 months of age, all of whom were clinically diagnosed with sacrococcygeal skin dimples, 57 of whom had FCs, and one who had a tethered cord. A total of 28 cases were followed up, and the ultrasound results showed that the cysts disappeared or did not change significantly.

The aforementioned literature shows that the presence of FCs is negatively correlated with age. With increasing age, FCs decrease and disappear on their own. It can be inferred that the incidence of fetal FCs is higher than that in infancy. However, there are few reports on prenatal diagnosis of FCs. Combined with the analysis of two cases of missed prenatal diagnosis in this group, we believe that the reason for the low prenatal detection rate of FCs is probably due to the lack of understanding of the abnormal prenatal ultrasound features and the scanning of the conus medullaris, which often only focuses on the position of its lower edge while ignoring whether there is an abnormal echo in the end spinal canal. Because FC lesions are small, they are prone to misdiagnosis. Additionally, for cysts close to the near field, insufficient ultrasound resolution or inappropriate gain adjustment are reasons for missed diagnoses. Furthermore, fetal position factors, such as the occipital posterior position and sacral posterior position may also make it difficult to observe the fetal spine and lead to misdiagnosis. MRI showed that the T2 signals of FCs was only slightly higher than that in the surrounding cerebrospinal fluid, which has no significant advantage over Ultrasound. However, MRI remains valuable in ruling out the possibility of other pathologies such as lipomas.

### 4.2 Differential diagnosis of FCs

**FIGURE 4:**
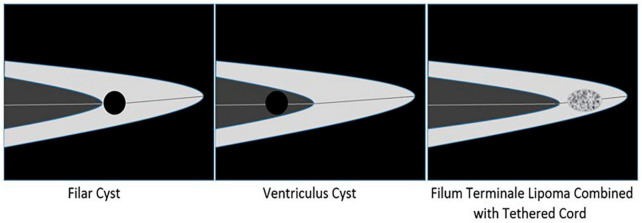
Schematic diagram of identification of Filar cyst, Ventriculus cyst, and Filum terminale lipoma combined with tethered cord.

Filar cysts should be differentiated from endometrial lipomas and end-chamber cysts. (1) Filum terminale lipoma: When fatty deposits in the terminale are tumor-like, they are called filum terminale lipomas or terminal hyperlipidosis (8). A lipoma terminale may involve the subdural or epidural filum terminale, and, unlike cysts that are anechoic, ultrasound findings are moderate or hyperechoic. Fatty degeneration can damage the spinal cord by external traction or excessive flexion and extension, leading to tethered cord syndrome (9). It can also be combined with other deformities, such as spina bifida and meningoceles, with neural tube insufficiency being the most common. This can also be considered a normal variation in the absence of concurrent abnormalities. (2) Ventricular cyst: The ventriculus terminalis (VT) is a cystic chamber located in the conus medullaris, surrounded by normal ependymal tissue and cerebrospinal fluid. If the end chamber is widened locally, it can manifest on ultrasound as a localized anechoic area at the center of the conus medullaris, which is called an end-chamber cyst (10). Most VTs are stable in size and disappear spontaneously. In comparison, filum-terminale cysts are located within the terminal filament of the conus medullaris. A simple schematic of the differentiation between FCs and the other two conditions is shown in Figure 4.

### 4.3 Limitations

The limitations of this study are as follows: (1) a pathological diagnosis was not done in this group; and (2) the sample size was small, and no other structural anomalies were combined; therefore, it is necessary to expand the sample size for subsequent research.

## 5 Conclusion

Isolated fetal FCs are easily misdiagnosed prenatally. A comprehensive and detailed ultrasound scan of the spinal canal at the end of the conus medullaris is helpful for the prenatal diagnosis and follow-up of FCs. Isolated FCs have a good prognosis and usually show no clinical symptoms. However, attention should still be paid to the presence of abnormalities such as lipoma terminale, tethered cord syndrome, and occult spina bifida.

## Data availability statement

The original contributions presented in the study are included in the article/Supplementary material, further inquiries can be directed to the corresponding author.

## Ethics statement

The studies involving humans were approved by the Ethics Committee of Chongqing Health Center for Women and Children. The studies were conducted in accordance with the local legislation and institutional requirements. The participants provided their written informed consent to participate in this study. Written informed consent was obtained from the individual(s) for the publication of any potentially identifiable images or data included in this article.

## Author contributions

SC: Writing – original draft, Writing – review and editing. PT: Data curation, Investigation, Writing – original draft. LM: Writing – review and editing.

## Supplementary material

Supplementary Figure 1 Sagittal T2 fetal MRI shows filar cyst (yellow arrow).
