# Supplementary figures and images for: Prenatal ultrasound diagnosis and prognosis of fetus with isolated filar cyst: a retrospective analysis
Source: Front Med (Lausanne). 2024 Jan 22;11:1304803. doi: 10.3389/fmed.2024.1304803 (PMC10839057; doi:10.3389/fmed.2024.1304803)

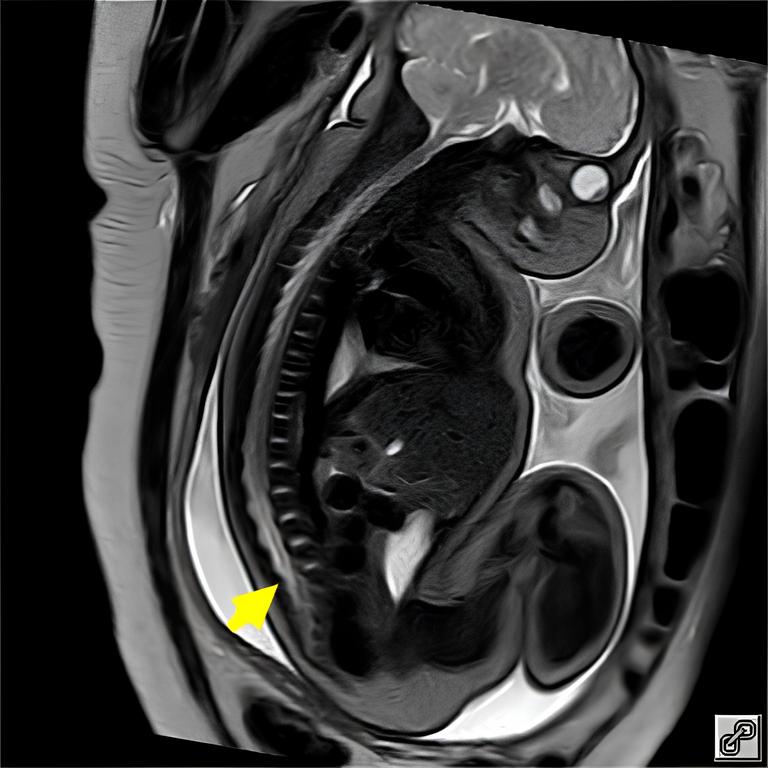

Supplement: Supplementary Figure 1 — Sagittal T2 fetal MRI shows filar cyst (yellow arrow). [file Image_1.JPEG]
